# The effectiveness of virtual reality in people with osteoporosis or osteopenia: a systematic review and meta-analysis of randomized controlled trials

**DOI:** 10.3389/fphys.2025.1612882

**Published:** 2025-07-02

**Authors:** Shunxia He, Shiqiu Dong, Xiaoguang Lin, Zhijie Wang, Yuzi Diao, Xiao Gao

**Affiliations:** ^1^ Heilongjiang University of Chinese Medicine, Harbin, China; ^2^ Heilongjiang Nursing College, Harbin, China; ^3^ Graduate School, Gachon University, Seongnam-si, Gyeonggi-do, Republic of Korea; ^4^ Shanxi Province Hospital of Traditional Chinese Medicine, Taiyuan, China; ^5^ Heilongjiang Vocational College of Winter Sports, Harbin, China; ^6^ The Fourth Affiliated Hospital of Heilongjiang University of Chinese Medicine, Harbin, China

**Keywords:** osteopenia, virtual reality, exercise, aging, osteoporosis

## Abstract

**Background:**

Osteoporosis is a global bone disease, and drug therapy carries the risk of side effects, requiring exploration of safe and effective alternative therapies. Virtual reality (VR) has shown potential in rehabilitation medicine, but its efficacy in the management of osteoporosis and osteopenia has not been systematically evaluated.

**Method:**

Using PubMed, Embase, the Cochrane Library, and Web of Science, we conducted a comprehensive database search to identify randomized controlled trials (RCTs) investigating the effects of VR on osteoporosis and bone loss. Trials published up to 30 March 2025 met our predefined inclusion and exclusion criteria. We extracted data, reviewed the literature. We assessed the methodological quality of the included trials and the certainty of the pooled evidence. Meta-analyses were performed using StataSE version 16.

**Results:**

Our meta-analysis included 216 patients from 5 RCTs. All cases included in our study adopted the non-immersive VR intervention approach. Femoral neck bone mineral density (BMD) (standardized mean difference [SMD] = 0.77, 95% confidence interval [CI] = 0.35–1.19, P < 0.0001, I^2^ = 0%), balance (SMD = 2.58, 95% CI = 1.10–4.05, P = 0.001, I^2^ = 91.2%) and mobility (SMD = 1.63, 95% CI = 0.14–3.13, P = 0.032, I^2^ = 93.7%) were improved compared to the control group. However, lumbar BMD (SMD = 0.39, 95% CI: −0.02, 0.80, P = 0.062, I^2^ = 0%) and quality of life (QOL) (SMD = 2.50, 95% CI: −2.15, 7.16, P = 0.292, I^2^ = 97.4%) were not significantly improved compared to the control group. None of the included studies reported adverse events or safety data.

**Conclusion:**

This systematic evaluation provides valuable evidence for the management of osteoporosis and osteopenia through VR. However, given the overall low and very low level of evidence, the results need to be treated with caution. In the future, VR may be a potential treatment for osteoporosis and osteopenia.

**Systematic Review Registration:**

https://www.crd.york.ac.uk/PROSPERO/view/CRD420251023178, PROSPERO, identifier CRD420251023178.

## 1 Introduction

Osteoporosis is a systemic skeletal disease characterized by decreased bone mass and deterioration of bone microarchitecture, with a core diagnostic criterion of bone mineral density (BMD) T-value ≤ −2.5 (Kanis, 1990; Rosen, 2018). Osteopenia is a transitional state between normal and osteoporotic BMD, with a T-value of −1.0 to −2.5 (Karaguzel and Holick, 2010). Osteopenia is often considered a precursor of osteoporosis, and may signal a further loss of BMD (Karaguzel and Holick, 2010). Aging and feminization are associated with osteoporosis, and women have a significantly increased risk of osteoporosis after menopause (Aspray and Hill, 2019; Fan et al., 2024). One study showed that the number of new cases of osteoporosis is expected to reach 41.5 million globally in 2019, and is expected to increase to 263.2 million cases between 2030 and 2034 (Zhu et al., 2023). Osteoporosis is a huge medical and economic burden in all regions of the world (Hopkins et al., 2016; Aziziyeh et al., 2019; Tatangelo et al., 2019). Currently in osteoporosis, medication reduces the risk of fracture and stimulates bone formation, increasing BMD (Reid and Billington, 2022). However, medication also has some side effects, which need to be treated with caution (Khan et al., 2017). Therefore, there is a need to develop alternative therapies that are safe, accessible and have a high level of participation.

In addition to pharmacological interventions, various nonpharmacological therapies have been shown to improve balance and mobility in patients with osteoporosis or osteopenia. For example, traditional physical therapy programs focusing on strength training, balance exercises, and aerobic exercise have been shown to have positive effects on physical function and quality of life in patients with osteoporosis and osteopenia (Chen et al., 2019). Similarly, exercises such as Tai Chi have gained recognition for their potential to enhance balance and reduce the risk of falls in older adults. These traditional interventions play a vital role in the management of osteoporosis and osteopenia (Li et al., 2004). However, they can face challenges in terms of patient engagement and compliance, especially in those who find the exercises monotonous or lack motivation.

Virtual Reality (VR) is a technology that provides multi-sensory interactive experiences through computer simulation of three-dimensional environments, and its core classifications include immersive and non-immersive (Prinz et al., 2023). In recent years, the application of VR in medicine has expanded from surgical training to rehabilitation, and has demonstrated unique advantages in neurorehabilitation and chronic pain management (Pourmand et al., 2017). VR-based rehabilitation is more conducive to the development of physical health than conventional rehabilitation, positively affecting recovery of aerobic function, balance, pain levels, psychological and motor function, in addition to improving patient motivation (Howard, 2017; de Araújo et al., 2019). However, as an emerging treatment modality, VR initially faced implementation barriers, including large financial investments, technical challenges, and operator training (Glegg and Levac, 2018; Chung et al., 2021; Sarkar et al., 2021). Based on the development of VR in rehabilitation, researchers have begun to explore its use in patients with osteoporosis or osteopenia, but the dispersed nature of the available evidence and the lack of systematic summarization have hindered the clinical translation process.

Preliminary clinical trials suggest that VR interventions are effective in improving balance function in patients with osteoporosis (Yilmaz and Kösehasanoğulları, 2024). A study of patients with postmenopausal osteoporosis showed that VR was effective in improving physical performance and quality of life (QOL) (Riaz et al., 2024b). In addition, Meta-analyses for other musculoskeletal disorders further support the efficacy of VR. Both non-immersive and immersive VR-assisted active training are effective in reducing back and neck pain symptoms (Lo et al., 2024). VR-based rehabilitation improves pain, motor function, and anxiety in total knee replacement patients within 1 month after surgery. However, no study has comprehensively evaluated the effects of VR on functional outcomes in patients with osteoporosis or osteopenia.

To fill this knowledge gap, this study aimed to integrate the existing evidence through systematic evaluation and Meta-analysis to provide an evidence-based basis for the clinical application of VR in the management of osteoporosis and osteopenia. These findings specifically focus on BMD, the effects of balance, mobility and QOL.

## 2 Methods

This systematic review and meta-analysis was conducted in accordance with the Preferred Reporting Items for Systematic Reviews and Meta-Analyses (PRISMA) guidelines (Moher et al., 2009). Comprehensive methodological details are outlined in Supplementary Table 1. The study protocol has been registered with International Prospective Register of Systematic Reviews (PROSPERO) under the identifier CRD420251023178.

### 2.1 Search strategy

We searched PubMed, Embase, Web of Science, and Cochrane databases from inception to 30 March 2025, using the terms “virtual reality,” “osteoporosis,” and “randomized controlled trial.” To minimize missed studies, we also reviewed the references of included studies. The search strategies for each database are detailed in Supplementary Table 2.

### 2.2 Eligibility criteria and study selection

Studies were included based on the PICOs criteria:

(1) Populations: Patients aged ≥18 years with a clinical diagnosis of osteoporosis or osteopenia (WHO Study Group, 1994);
(2) Interventions: VR;
(3) Comparator: Non-VR interventions such as active control (traditional training); passive control (health education, walking or placebo);
(4) Outcomes: BMD, Balance, Mobility, and Quality of life (QOL);
(5) Study Design: Randomized controlled trials (RCTs).

Studies were excluded if they were (1) conference abstracts, (2) animal studies, (3) unpublished papers, (4) non-English language studies, or (5) ongoing studies or protocols. Two independent reviewers (SXH and XGL) conducted parallel title/abstract screening using predefined eligibility criteria. Articles meeting preliminary inclusion thresholds underwent subsequent full-text evaluation by both reviewers. Inter-rater discrepancies were resolved via consensus-based adjudication involving a senior researcher (XGL). Search results were imported into EndNote version X9 (Thomson Research Software, Stamford, CT, United States).

### 2.3 Data extraction

Extracted data included study characteristics (authors’ names, year of publication, and study location), participant details (sample size and mean age), intervention characteristics (intervention specifics for both groups, frequency, and duration), and outcome metrics. For missing data within the studies, we chose to contact the corresponding author via email. If data could not be obtained, we excluded the studies. We uniformly transformed the data into means and standard deviations to summarize the results. Two reviewers (SXH and XGL) independently performed data extraction, with verification by a third reviewer (XG). We calculated Cohen’s kappa coefficients (κ) for a randomly selected subset of 20% of the included studies (n = 1/5) to validate the consistency of screening by two independent researchers (Landis and Koch, 1977).

### 2.4 Quality assessment and certainty of evidence

The methodological quality of the studies was assessed by using the Physiotherapy Evidence Database (PEDro) scale (Cashin and McAuley, 2020). Higher scores (lowest score = 0; highest score = 10) indicate better methodological quality on the 11-item PEDro scale. To classify studies according to their quality, the following cut-off points were proposed: excellent (9–10), good (6–8), fair (4–5), and poor (≤3). The Grading of Recommendations Assessment, Development, and Evaluation (GRADE) methodology was used to assess the certainty of evidence, with rankings ranging from high to very low (Meader et al., 2014).

### 2.5 Data analysis

Data from included studies were converted to means and standard deviations for summary. Graphical data were extracted via GetData Graph Digitizer (v2.22) for numerical conversion. Random-effects models were used to summarize each outcome, reporting the standard mean difference (SMD) and 95% confidence interval (CI) (Moher et al., 2009). Heterogeneity quantification employed Cochran’s Q statistic supplemented by I^2^ metrics, applying conventional interpretation thresholds: I^2^ <50% (low), 50%–75% (moderate), and >75% (substantial). A P-value <0.05 was considered statistically significant.

Sensitivity analyses were performed using a stepwise exclusion of single studies to ensure the robustness of findings. Subgroup analyses and meta-regression were performed to explore heterogeneity according to the type of control group (Berkey et al., 1995). Data analysis was performed using StataSE (version 16. 0; Stata Corp LP, College Station, Texas, The United States of America).

## 3 Results

### 3.1 Search result

**FIGURE 1:**
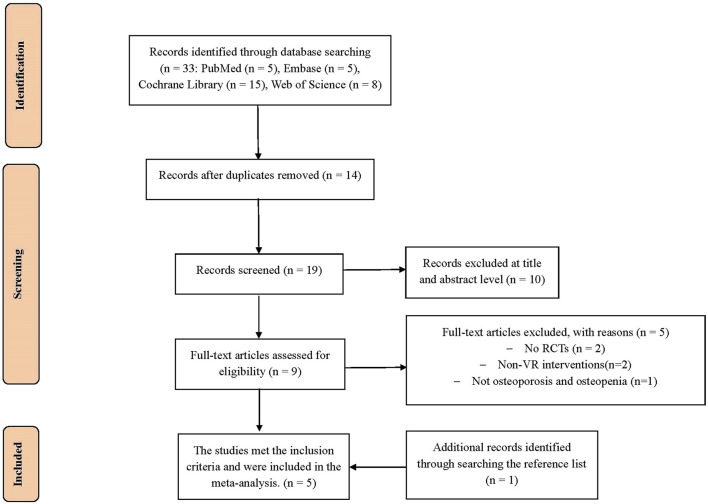
PRISMA flow chart for the study selection.

From the databases used for the initial search, 33 potentially relevant studies were identified (PubMed, n = 5; Embase, n = 5; Cochrane Library, n = 15; Web of Science, n = 8). After de-duplication, 19 studies were screened on the basis of title and abstract. Subsequently, after deleting 15 studies, 4 studies remained for meta-analysis. In addition, 1 study was screened by manually searching the reference list. Finally, a total of 5 studies (Gilani et al., 2023; Zhao et al., 2023; Riaz et al., 2024a; Riaz et al., 2024b; Yilmaz and Kösehasanoğulları, 2024) were included for data summarization (Figure 1).

### 3.2 Characteristics of included studies

Inter-rater reliability of data extraction was assessed using the Cohen’s kappa coefficient (κ) for a 20% sample of randomized studies. κ value of 0.82 (95% CI 0.72–0.92) reflects a high degree of agreement among reviewers, ensuring the robustness of the data extraction process. All five studies involved a total of 216 patients. The five studies were from Pakistan, China, Iran and Turkey. The mean age of the patients in the experimental group ranged from 58.3 to 72.2 years, and the mean age of the patients in the control group ranged from 58 to 73.4 years. The VR sessions ranged from 45 to 51 min each, three times a week, for 6–48 weeks. The control group received conventional training, home exercise, or walking, while the experimental group had VR interventions. None of the studies reported follow-up data. Table 1 summarizes the characteristics of the included studies.

**TABLE 1:** Characteristics of included articles.

Study	Country	Design	Diagnosis	Inclusion criteria	Experimental group				Control group				Outcome
					Sample size	Age (year)	Intervention	Time, frequency	Sample size	Age (year)	Intervention	Time, frequency	
Riaz et al. (2024a) (1)	Pakistan	RCT	osteopenia	Lumbar or femoral T-scores between −1 and −2.5	22	58.3 ± 5.1	VR provided by Xbox Kinect; Walking Outdoors	VR: 45 min per session, 3 times per week for 24 weeks; Walking outdoor: 30 min per day	21	58.0 ± 5.5	Walking Outdoors	30 min per day	BMD
Riaz et al. (2024b) (2)	Pakistan	RCT	osteopenia	Lumbar or femoral T-scores between −1 and −2.5	22	58.3 ± 5.2	VR provided by Xbox Kinect; Walking Outdoors	VR: 45 min per session, 3 times per week for 24 weeks; Walking outdoor: 30 min per day	21	58.0 ± 5.6	Walking Outdoors	30 min per day	Balance, Mobility, QOL
Yilmaz and Kösehasanoğulları (2024)	Turkey	RCT	osteoporosis	Lumbar spine and femoral neck T score < -2.5	30	67 ± 10.6	VR provided by Nintendo Wii-based	3 times per week for 12 weeks	30	68 ± 9.1	Traditional training (strength training, aerobic training, and balance exercises)	3 times per week for 12 weeks	Balance, Mobility
Gilani et al. (2023)	Iran	RCT	osteoporosis	Lumbar spine and femoral neck T score < -2.5	10	58.5 ± 6.2	VR provided by Xbox Kinect	51 min per day, 3 times per week for 6 weeks	10	59.4 ± 4.7	Traditional training (strength training and balance training)	45–50 min per day, 3 times per week for 6 weeks	Mobility, QOL
Zhao et al. (2023)	China	RCT	osteoporosis	Lumbar spine and femoral neck T score < -2.5	25	72.2 ± 3.6	VR rehabilitation training system	50 min per session, 3 times per week for 12 months	25	73.4 ± 3.3	Traditional training (core muscle training, lower extremity muscle strength training, balance training and gait function training)	50 min per session, 3 times per week for 12 months	Balance, Mobility, BMD

RCTs, randomized controlled trials; VR, virtual reality; QOL, quality of life; BMD, bone mineral density.

Data are shown as mean standard deviation where appropriate.

### 3.3 Risk of bias assessment and certainty of evidence

To assess the quality of the five included studies, the PEDro scale was used, which ranged from 5 to 8. Four (Gilani et al., 2023; Zhao et al., 2023; Riaz et al., 2024a; 2024b) of the five studies were considered to be of good quality and one study (Yilmaz and Kösehasanoğulları, 2024) was considered to be of fair quality. The primary methodological flaws in our included studies were: lack of intention-to-treat analysis (0/5), inadequate therapist blinding (1/5), and absence of participant blinding (1/5). Table 2 shows the PEDro scores of the included studies. Ratings using the GRADE methodology for all outcome measurements were inconsistent and ranged from low to very low certainty in Supplementary Table 3. Evidence certainty was downgraded by one level for serious concerns in two domains: risk of bias and imprecision.

**TABLE 2:** The methodological quality of included studies on the PEDro scale.

Study	Items of PEDro scale											Total scores
	1	2	3	4	5	6	7	8	9	10	11	
Riaz et al. (2024a) (1)	Yes	1	1	1	1	0	1	0	0	1	1	7
Riaz et al. (2024b) (2)	Yes	1	1	1	1	0	1	0	0	1	1	7
Yilmaz and Kösehasanoğulları (2024)	Yes	1	0	1	0	0	0	1	0	1	1	5
Gilani et al. (2023)	Yes	1	1	1	0	1	1	1	0	1	1	8
Zhao et al. (2023)	Yes	1	0	1	1	0	0	1	0	1	1	6

Items of the PEDro, scale: 1 = specified eligibility criteria (yes/no); 2 = random allocation; 3 = concealed allocation; 4 = comparability at baseline; 5, blinded subjects; 6, blinded therapists; 7, blinded assessors; 8, sufficient follow-up; 9, intention-to-treat analysis 10 = comparison between groups; 11 = point estimates and variability. For terms 2–11:1, the corresponding criterion is satisfied; 0, the criterion is not satisfied.

### 3.4 Outcomes synthesis

This section summarizes the results of VR acting on the osteoporotic population. At least two or more included studies reported on BMD, balance, mobility and QOL. Details of the tools involved in the included studies can be found in Supplementary Table 4.

#### 3.4.1 BMD

**FIGURE 2:**
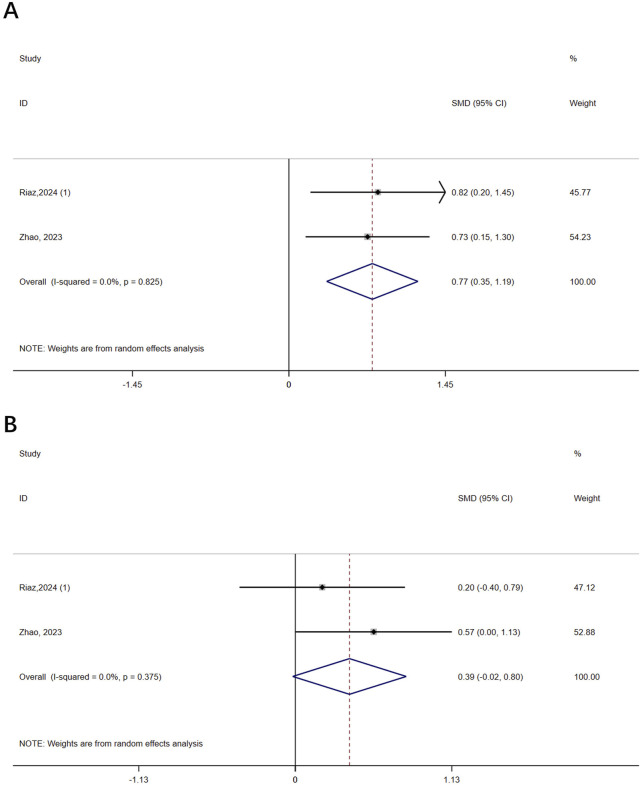
Forest plot of the effect of virtual reality on bone mineral density (BMD). **(A)** Femoral neck BMD; **(B)** Lumbar spine BMD.

Two studies (n = 93) assessed the severity of osteoporosis in patients with osteoporosis using BMD. The results showed that VR improved femoral neck BMD (SMD = 0.77, 95% CI: 0.35, 1.19, P < 0.0001, I^2^ = 0%) but not lumbar spine BMD (SMD = 0.39, 95% CI: −0.02, 0.80, P = 0.062, I^2^ = 0%) compared to controls (Figure 2). The certainty of evidence was low for both (Supplementary Table 3).

#### 3.4.2 Balance

**FIGURE 3:**
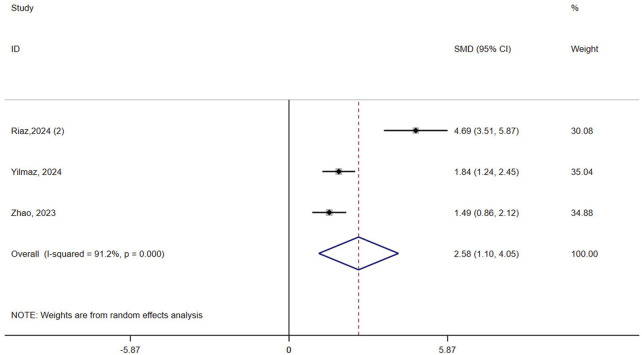
Forest plot of the effect of virtual reality on balance.

Three studies (n = 153) assessed balance in osteoporotic patients. The results showed that VR improved balance compared to controls (SMD = 2.58, 95% CI: 1.10, 4.05, P = 0.001, I^2^ = 91.2%) (Figure 3). The certainty of the evidence was very low (Supplementary Table 3). Robust result was obtained by excluding one Riaz study from the sensitivity analysis and reducing heterogeneity to 0% (Supplementary Figure 1A). Subgroup analysis based on the intervention in the control group revealed a greater advantage of VR in the passive control (SMD = 4.69, 95% CI: 3.51, 5.87, P < 0.0001) compared to the active control (SMD = 1.67, 95% CI: 1.24, 2.11, P < 0.0001, I^2^ = 0%) (Supplementary Figure 2A). The meta-regression results showed that the type of control group was a contributing factor to heterogeneity (P = 0.043).

#### 3.4.3 Mobility

**FIGURE 4:**
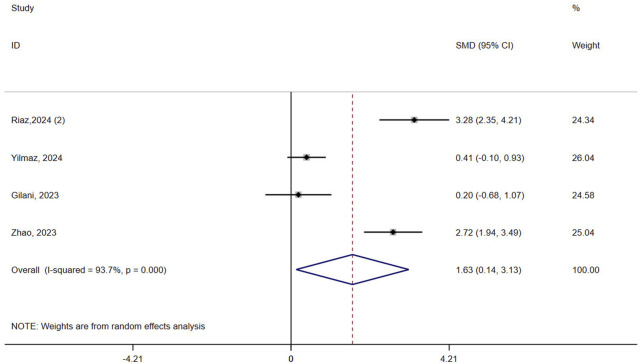
Forest plot of the effect of virtual reality on mobility.

Four studies (n = 173) assessed mobility in patients with osteoporosis. Results showed that VR improved mobility compared to controls (SMD = 1.63, 95% CI: 0.14, 3.13, P = 0.032, I^2^ = 93.7%) (Figure 4). The certainty of evidence was very low (Supplementary Table 3). Sensitivity analyses did not reduce the heterogeneity of result (>90%) (Supplementary Figure 1B). Subgroup analysis based on the intervention in the control group revealed a greater advantage of VR in the passive control (SMD = 3.28, 95% CI: 2.35, 4.21, P < 0.0001) compared to the active control (SMD = 1.10, 95% CI: −0.41, 2.61, P = 0.151, I^2^ = 92.6%) (Supplementary Figure 2B). The meta-regression result showed that the type of control group was not a contributing factor to heterogeneity (P = 0.314).

#### 3.4.4 QOL

**FIGURE 5:**
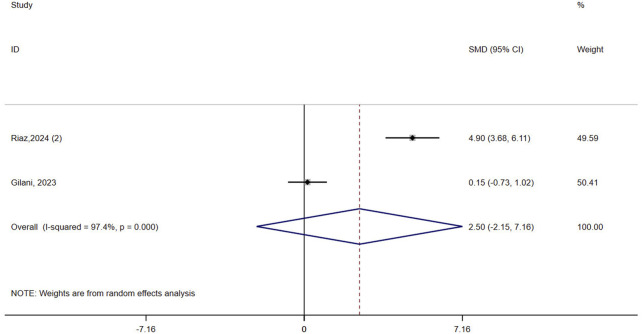
Forest plot of the effect of virtual reality on quality of life (QOL).

Two studies (n = 63) assessed QOL in patients with osteoporosis. The result showed that VR did not improve QOL compared to controls (SMD = 2.50, 95% CI: −2.15, 7.16, P = 0.292, I^2^ = 97.4%) (Figure 5 **)**. The certainty of evidence was very low (Supplementary Table 3).

## 4 Discussion

This study is the first meta-analysis to assess the effectiveness of a VR intervention on functional outcomes in patients with osteoporosis or osteopenia. The results showed that VR improved femoral neck BMD but had no significant effect on lumbar spine BMD. In terms of balance function and mobility, the VR intervention group was better than the control group, but the improvement in QOL did not reach statistical significance. Regarding the quality of the literature, four of the five included papers were of high quality. Sensitivity analyses further confirmed the robustness of the results for balance function, but heterogeneity in mobility remained high. GRADE scores for all evidence ranged from low to very low. This stems from risk of bias and imprecision, mainly because the evidence includes studies that are at risk of bias. In addition, the total sample size for each piece of evidence was low. Therefore, results need to be interpreted with caution.

The results of this study correlate with previous trends in the use of VR in rehabilitation medicine. VR highlights the importance of integrating games into rehabilitation programs as they allow observation of movement and control of activity levels. Games using Kinect technology have been shown to be highly effective in treating chronic diseases (Tarakci et al., 2016). The improvement of balance function and mobility in osteoporosis and osteopenia by VR echoes research in the field of neurorehabilitation, which may involve enhanced proprioceptive input and motor learning efficiency (Cano Porras et al., 2018; Zhang et al., 2021). BMD can be used as an indicator to define the severity of osteoporosis and the effectiveness of treatment (Löffler et al., 2020). A study has shown that VR biofeedback systems greatly enhance tissue regeneration, exercise efficacy and participation in training (Wang, 2024). Improvements in femoral neck BMD suggest that VR may stimulate local bone remodeling in the lower limb through mechanical loading. However, lumbar spine BMD did not significantly improve. This may be due to the fact that the VR rehabilitation programs focused on lower limb training and less on trunk training, which could be validated by adjusting the protocol in the future. On the other hand, nonsignificant lumbar BMD results may reflect inadequate statistical power rather than true biological invalidity. Small sample sizes are prone to Type II errors, resulting in false negatives (Mittendorf et al., 1995). In addition, negative QOL results may be influenced by multidimensional factors, such as psychological state and sociability, and existing studies have not targeted interventions in these areas (Gilani et al., 2023; Zhao et al., 2023).

Confounding factors can increase the heterogeneity of results, which may also be responsible for biased results. In the balanced results, subgroup analyses, meta-regression, and sensitivity analyses showed that VR showed better efficacy and decreased outcome heterogeneity in passive controls. The significant advantage of VR in passive controls may stem from the low intensity of the control measures, while the effectiveness of the active controls themselves may have diluted the effect of VR. Notably, the dominance of VR in the passive control group may reflect its potential as a stand-alone intervention, particularly in scenarios where structured rehabilitation resources are lacking. In addition, Mobility showed less effect of control group type on heterogeneity in all tests. The high heterogeneity in mobility and QOL may be related to differences in assessment tools and small sample sizes. Heterogeneity may also come from VR type. However, the types of VRs included in the study were all non-immersive VRs, and it was not possible to identify the effect of VR type on the results from the nature of the VRs. On the other hand, it is also possible that the heterogeneity originated from the systems of different non-immersive VRs, due to differences in the design of each system (Tarakci et al., 2016; Kumar et al., 2017). It is also possible that the heterogeneity was influenced by the duration of the intervention, which ranged from a minimum of 6 weeks to a maximum of 12 months for the included studies using VR. Subgroup analyses were not possible due to the small number of studies with the same factors. Future studies need to standardize outcome assessment methods and expand sample sizes to reduce confounding bias.

The mechanism of action of VR in osteoporosis management may involve multi-pathway synergies. Firstly, VR training optimizes neuromuscular coordination by inducing motor control and learning in an immersive virtual environment where patients combine visual, motor and haptic signals to perform high-quality exercises (Maden et al., 2025). Secondly, the real-time visual feedback provided by VR may improve proprioceptive integration, reduce the risk of falls and indirectly protect the bone microstructure (Raffegeau et al., 2023). In addition, unlike the repetitive nature of traditional training, VR training is full of motivation and fun, which can significantly improve patient compliance (Riaz et al., 2024a). VR can guide patients to make postural changes during training, thereby increasing localized loads on the body. However, the negative results of lumbar spine BMD suggest that the intensity of mechanical stimulation of the spine by VR may be insufficient and further optimization of the exercise programs design is required.

When considering incorporating VR interventions into routine clinical practice, it is important to weigh these costs against the potential benefits of VR interventions. Although the initial investment in VR technology is substantial, the long-term cost-effectiveness may be greater because reduced healthcare costs are associated with improved patient outcomes (e.g., fewer fractures and hospitalizations). In addition, as VR technology becomes more popular and market competition increases, the costs of VR interventions may decrease (Geraets et al., 2021). The feasibility of implementing VR interventions in a clinical setting is another key consideration. VR requires specialized equipment and trained personnel to operate and maintain the system. However, as technology advances, VR systems are becoming more user-friendly and accessible (Ciccone et al., 2023). Furthermore, it is also important to consider whether healthcare providers have access to technical support and training to ensure smooth implementation of VR interventions (Hood et al., 2021; Iqbal et al., 2024). Patient compliance is a key factor in the success of any intervention, and VR is no exception. The engaging and interactive nature of VR can enhance patient motivation and compliance. One study has shown that patients are more likely to complete VR exercises than traditional rehabilitation exercises due to the fun and immersive experience VR provides (Cano Porras et al., 2018). It is also critical to select appropriate VR content and adjust the length and intensity of VR sessions to minimize these side effects (Mao et al., 2021).

There are some limitations to this study. Firstly, the small number of included studies and limited sample size may reduce statistical validity. The small number of included studies also resulted in an inability to detect publication bias. Second, the high heterogeneity of the intervention protocols, such as differences in the type of VR equipment and training duration, limits the generalizability of the findings. Finally, there was a lack of long-term follow-up data to assess the persistence effect of the results. Future multicenter large-sample RCTs and in-depth investigation of the mechanisms of VR in osteoporosis and bone loss are needed. In addition, VR combined with artificial intelligence to adjust the intensity or in combination with anti-bone resorption drugs may be a new direction to optimize the efficacy. Despite the positive effects of VR on osteoporosis and bone loss, certain uncertainties remain and further research is needed. The results of the meta-analyses showed only effectiveness. However, the adverse effects and safety of VR remain unknown. More comprehensive studies of VR therapy are necessary in the future to more fully understand its effects. In addition, future research also needs to verify different VR types and different VR training parameters. In addition to verifying the effectiveness of VR, it is also possible to compare several commonly used treatment parameters in previous studies and establish standards for VR treatment of osteoporosis and osteopenia.

## 5 Conclusion

VR-based rehabilitation is a novel and promising treatment modality that is increasingly being implemented in clinical settings. This study is the first to assess the impact of VR intervention on functional outcomes in patients with osteoporosis or bone loss through systematic evaluation and meta-analysis. Given the overall low and very low level of evidence, the results need to be treated with caution. While these results suggest that VR has potential as an adjunctive therapy, its clinical application is still in the research phase. In addition, no study has conclusively demonstrated the clinical safety of virtual reality interventions for individuals with osteoporosis or osteopenia. The sustained effects, safety and potential side effects of VR still need to be further evaluated. Future RCTs need to focus on extending follow-up, exploring bone remodeling effects and stratifying the baseline population.

## Data Availability

The original contributions presented in the study are included in the article/Supplementary Material, further inquiries can be directed to the corresponding author.
