# Supplementary material for: The effectiveness of virtual reality in people with osteoporosis or osteopenia: a systematic review and meta-analysis of randomized controlled trials
Source: Front Physiol. 2025 Jul 2;16:1612882. doi: 10.3389/fphys.2025.1612882 (PMC12263551; doi:10.3389/fphys.2025.1612882)
Supplement: Supplementary file 1 [file Supplementaryfile1.docx]

**Supplementary Materials**

**Supplementary Table 1.** PRISMA 2009 checklist

**Supplementary Table 2.** Search strategy from database inception to March 30, 2025 **Supplementary Table 3.** The results of GRADE assessment of the evidence certainty **Supplementary Table 4**. Interpretation of assessment tools used in the study of pooled results

**Supplementary Fig. 1.** Sensitivity analysis of pooled results

**Supplementary Fig. 2.** Pooled results of subgroup analysis based on interventions of control group.

**Supplementary Table 1.** PRISMA 2009 checklist

| **Section/topic** | **#** | **Checklist item** | **Reported #** |
| --- | --- | --- | --- |
| **TITLE** | | |  |
| Title | 1 | Identify the report as a systematic review, meta-analysis, or both. | Title Page |
| **ABSTRACT** | | |  |
| Structured summary | 2 | Provide a structured summary including, as applicable: background; objectives; data sources; study eligibility criteria, participants, and interventions; study appraisal and synthesis methods; results; limitations; conclusions and implications of key findings;  systematic review registration number. | Title Page |
| **INTRODUCTION** | | |  |
| Rationale | 3 | Describe the rationale for the review in the context of what is already known. | Introduction Paragraphs 1-4 |
| Objectives | 4 | Provide an explicit statement of questions being addressed with reference to participants, interventions, comparisons, outcomes, and study design (PICOS). | Introduction Paragraphs 5 |
| **METHODS** | | |  |
| Protocol and registration | 5 | Indicate if a review protocol exists, if and where it can be accessed (e.g., Web  address), and, if available, provide registration information including registration number. | Method |
| Eligibility criteria | 6 | Specify study characteristics (e.g., PICOS, length of follow-up) and report  characteristics (e.g., years considered, language, publication status) used as criteria for eligibility, giving rationale. | Method 2.2 |

| Information sources | 7 | Describe all information sources (e.g., databases with dates of coverage, contact with study authors to identify additional studies) in the search and date last searched. | Method 2.1 |
| --- | --- | --- | --- |
| Search | 8 | Present full electronic search strategy for at least one database, including any limits used, such that it could be repeated. | Method 2.1 |
| Study selection | 9 | State the process for selecting studies (i.e., screening, eligibility, included in systematic review, and, if applicable, included in the meta-analysis). | Method 2.3 |
| Data collection process | 10 | Describe method of data extraction from reports (e.g., piloted forms, independently, in duplicate) and any processes for obtaining and confirming data from investigators. | Method 2.3 |
| Data items | 11 | List and define all variables for which data were sought (e.g., PICOS, funding sources) and any assumptions and simplifications made. | Method 2.2 |
| Risk of bias in individual studies | 12 | Describe methods used for assessing risk of bias of individual studies (including specification of whether this was done at the study or outcome level), and how this information is to be used in any data synthesis. | Method 2.4 |
| Summary measures | 13 | State the principal summary measures (e.g., risk ratio, difference in means). | Method 2.5 |
| Synthesis of results | 14 | Describe the methods of handling data and combining results of studies, if done, including measures of consistency (e.g., I2) for each meta-analysis. | Method 2.5 |
| Risk of bias across studies | 15 | Specify any assessment of risk of bias that may affect the cumulative evidence (e.g., publication bias, selective reporting within studies). | NA: Bias in measurement due to low number of included studies |
| Additional analyses | 16 | Describe methods of additional analyses (e.g., sensitivity or subgroup analyses, meta-regression), if done, indicating which were pre-specified. | Method 2.5 |

| **Section/topic** | **#** | **Checklist item** | **Reported on page #** |
| --- | --- | --- | --- |
| **RESULTS** | | |  |
| Study selection | 17 | Give numbers of studies screened, assessed for eligibility, and included in the review, with reasons for exclusions at each stage, ideally with a flow diagram. | Result 3.1 |
| Study characteristics | 18 | For each study, present characteristics for which data were extracted (e.g., study size, PICOS, follow-up period) and provide the citations. | Result 3.2 |
| Risk of bias within studies | 19 | Present data on risk of bias of each study and, if available, any outcome level assessment (see item 12). | Result 3.3 |
| Results of individual studies | 20 | For all outcomes considered (benefits or harms), present, for each study: (a) simple summary data for each intervention group (b) effect estimates and confidence intervals, ideally with a forest plot. | Result 3.4 |
| Synthesis of results | 21 | Present results of each meta-analysis done, including confidence intervals and measures of consistency. | Result 3.4 |
| Risk of bias across studies | 22 | Present results of any assessment of risk of bias across studies (see Item 15). | Result 3.3 |
| Additional analysis | 23 | Give results of additional analyses, if done (e.g., sensitivity or subgroup analyses, meta-regression [see Item 16]). | Result 3.4 |
| **DISCUSSION** | | |  |
| Summary of evidence | 24 | Summarize the main findings including the strength of evidence for each main outcome; consider their relevance to key groups (e.g., healthcare providers, users, and policy makers). | Discussion Paragraphs 1-5 |

| Limitations | 25 | Discuss limitations at study and outcome level (e.g., risk of bias), and at review-level (e.g., incomplete retrieval of identified research, reporting bias). | Discussion Paragraphs 6 |
| --- | --- | --- | --- |
| Conclusions | 26 | Provide a general interpretation of the results in the context of other evidence, and implications for future research. | Conclusion |
| **FUNDING** | | |  |
| Funding | 27 | Describe sources of funding for the systematic review and other support (e.g., supply of data); role of funders for the systematic review. | Funding |

From: Moher, D., Liberati, A., Tetzlaff, J., Altman, D. G., & PRISMA Group. Preferred reporting items for systematic reviews and meta-analyses: the PRISMA statement. Ann Intern Med 151, 264–269, W64 (2009).

**Supplementary Table 2.** Search strategy from database inception to March 30, 2025

| **Database** | **Search term** | **Results** |
| --- | --- | --- |
| PubMed | (((((((((Virtual Reality[MeSH Terms]) OR (Reality, Virtual[Title/Abstract])) OR (Virtual Realit*, Educational[Title/Abstract])) OR (Educational Virtual Realit*[Title/Abstract])) OR (Reality, Educational Virtual[Title/Abstract])) OR (Virtual Reality, Instructional[Title/Abstract])) OR (Instructional Virtual Realit*[Title/Abstract])) OR (Realit*, Instructional Virtual[Title/Abstract])) AND ((((((((((((osteoporosis[MeSH Terms]) OR (Osteoporos*[Title/Abstract])) OR (Age-Related Osteoporos*[Title/Abstract])) OR (Osteoporoses, Age- Related[Title/Abstract])) OR (Bone Loss, Age-Related[Title/Abstract])) OR (Age-Related Bone Loss*[Title/Abstract])) OR (Bone Loss*, Age Related[Title/Abstract])) OR (Osteoporos*, Senile[Title/Abstract])) OR (Senile Osteoporos*[Title/Abstract])) OR (Osteoporosis, Post Traumatic[Title/Abstract])) OR (Post-Traumatic Osteoporos*[Title/Abstract])) OR (Osteopenia[Title/Abstract])) OR (Osteoporo*[Title/Abstract]))) AND (((((((Randomized Controlled Trials as Topic[MeSH Terms]) OR (randomized[Title/Abstract])) OR (randomised[Title/Abstract])) OR (randomization[Title/Abstract])) OR (random allocation[Title/Abstract])) OR  (placebo[Title/Abstract])) OR (Trial*[Title/Abstract])) | 5 |
| Embase | ('virtual reality'/exp/mj OR 'reality, virtual':ab,ti OR 'virtual realit*, educational':ab,ti OR 'educational virtual realit*':ab,ti OR 'reality, educational virtual':ab,ti OR 'virtual reality, instructional':ab,ti OR 'instructional virtual realit*':ab,ti OR 'realit*, instructional virtual':ab,ti) AND ('osteoporosis'/exp/mj OR osteoporos*:ab,ti OR 'age-related osteoporos*':ab,ti OR 'osteoporoses, age-related':ab,ti OR 'bone loss, age-related':ab,ti OR 'age-related bone loss*':ab,ti OR 'bone loss*, age related':ab,ti OR 'osteoporos*, senile':ab,ti OR 'senile osteoporos*':ab,ti OR 'osteoporosis, post traumatic':ab,ti OR 'post-traumatic osteoporos*':ab,ti OR ‘Osteopenia':ab,ti OR osteoporo*:ab,ti) AND ('randomized controlled trial'/exp/mj AND topic OR randomized:ab,ti OR randomised:ab,ti OR  randomization:ab,ti OR 'random allocation':ab,ti OR placebo:ab,ti OR trial*:ab,ti) | 5 |
| Cochrane Library | (MeSH descriptor: [Virtual Reality] explode all trees OR (Virtual Reality):ti,ab,kw OR (Reality, Virtual):ti,ab,kw OR  (Virtual Realit*, Educational):ti,ab,kw OR (Educational Virtual Realit*):ti,ab,kw OR (Reality, Educational | 15 |

|  | Virtual):ti,ab,kw OR (Virtual Reality, Instructional):ti,ab,kw OR (Instructional Virtual Realit*):ti,ab,kw OR (Realit*, Instructional Virtual):ti,ab,kw) AND (MeSH descriptor: [Osteoporosis] explode all trees OR (Osteoporos*):ti,ab,kw OR (Age-Related Osteoporos*):ti,ab,kw OR (Osteoporoses, Age-Related):ti,ab,kw OR (Bone Loss, Age- Related):ti,ab,kw OR (Age-Related Bone Loss*):ti,ab,kw OR (Bone Loss*, Age Related):ti,ab,kw OR (Osteoporos*, Senile):ti,ab,kw OR (Senile Osteoporos*):ti,ab,kw OR (Osteoporosis, Post Traumatic):ti,ab,kw OR (Post-Traumatic Osteoporos*):ti,ab,kw OR (Osteopenia):ti,ab,kw) OR (Osteoporo*):ti,ab,kw) AND (MeSH descriptor: [Randomized Controlled Trials as Topic] explode all trees OR (randomized):ti,ab,kw OR (randomised):ti,ab,kw OR  (randomization):ti,ab,kw OR (random allocation):ti,ab,kw OR (placebo):ti,ab,kw OR (Trial*):ti,ab,kw) |  |
| --- | --- | --- |
| Web of Science | (Virtual reality (Topic) or Reality, Virtual (Topic) or Virtual Realit*, Educational (Topic) or Educational Virtual Realit* (Topic) or Reality, Educational Virtual (Topic) or Virtual Reality, Instructional (Topic) or Instructional Virtual Realit* (Topic) or Realit*, Instructional Virtual (Topic) and Preprint Citation Index (Exclude – Database)) AND (osteoporosis (Topic) or Osteoporos* (Topic) or Age-Related Osteoporos* (Topic) or Osteoporoses, Age-Related (Topic) or Bone Loss, Age-Related (Topic) or Age-Related Bone Loss* (Topic) or Bone Loss*, Age Related (Topic) or Osteoporos*, Senile (Topic) or Senile Osteoporos* (Topic) or Osteoporosis, Post Traumatic (Topic) or Post- Traumatic Osteoporos* (Topic) OR (Osteopenia) (Topic) or Osteoporo* (Topic) and Preprint Citation Index (Exclude – Database)) AND (Randomized Controlled Trials as Topic (Topic) or randomized (Topic) or randomised (Topic) or randomization (Topic) or random allocation (Topic) or placebo (Topic) or Trial* (Topic) and Preprint Citation Index  (Exclude – Database)) | 8 |
| **Total** |  | 33 |

**Supplementary Table 3.** The results of GRADE assessment of the evidence certainty

| Outcomes | Study Design | Risk of Bias | Inconsistency | Indirectness | Imprecision | Publication bias | Other Consideration | Numbers of  Participants | | Effect size ( 95% CI ) | Quality |
| --- | --- | --- | --- | --- | --- | --- | --- | --- | --- | --- | --- |
|  |  |  |  |  |  |  |  | EG | CG |  |  |
| Femoral neck  BMD | RCTs | serious^a^ | no | no | Serious^d^ | no | no | 47 | 46 | 0.77 (0.35 to 1.19) | Low |
| Lumbar spine  BMD | RCTs | serious^a^ | no | no | Serious^d^ | no | no | 47 | 46 | 0.39 (-0.02 to 0.80) | Low |
| Balance | RCTs | serious^a^ | serious^b^ | no | Serious^d^ | no | no | 77 | 76 | 2.58 (1.10 to 4.05) | Very  Low |
| Mobility | RCTs | serious^a^ | serious^b^ | no | Serious^d^ | no | no | 87 | 86 | 1.63 (0.14 to 3.13) | Very  Low |
| QOL | RCTs | serious^a^ | serious^b^ | no | Serious^d^ | no | no | 32 | 31 | 2.50 (-2.15 to 7.16) | Very  Low |

**Abbreviation:** GRADE: Grading of Recommendations Assessment, Development and Evaluation EG: experimental group; CG: control group; RCTs: randomized controlled trials; QOL: Quality of Life.

**Criteria:** a Downgraded by one level for risk of bias;

b Downgraded by one level for inconsistency: Substantial heterogeneity is seen between studies (I²≥50%).

c Downgraded by one level for indirectness: Studies in different contexts have participants, interventions, or results that are not consistent with the actual question.

d Downgraded by one level for imprecision: Continuous variable events < 400.

e Downgraded by one level for publication bias: Contains unpublished studies; effect size overestimated or underestimated.

f Upgraded by one level for large effect size according to Grade standards：Effect size ＞3.

**Supplementary Table 4.** Interpretation of assessment tools used in the study of pooled results

| Assessment scales | Interpretations |
| --- | --- |
| TUG(1) | TUG test is a reliable, cost-effective, safe and time-efficient method of assessing overall functional mobility. The test requires the subject to stand up from a chair, walk forward 3 meters, turn around, walk back to the chair and sit down. The test is timed from the ‘start’ command until  the subject sits down. |
| BBS(2) | .The BBS is a standardized balance function assessment tool widely used in clinical practice to assess an individual's balance and co-ordination abilities. The BBS scale evaluates the patient's ability to actively shift the center of gravity by observing a variety of functional activities, providing a comprehensive examination of the patient's dynamic and static balance in both the  sitting and standing positions. |
| FRT(3) | The FRT is a simple and quick functional testing tool used to assess an individual's balance and fall risk. The test assesses balance by measuring the maximum distance an individual can reach forward without losing balance. The FRT scale is easy to administer and does not require complex  equipment, making it widely used in clinical and research settings. |
| ECOS-16(4) | The ECOS-16 is a simplified health-related quality of life questionnaire specifically designed to assess quality of life in postmenopausal women with osteoporosis. |
| Qualeffo-41 questionnaire(5) | The QUALEFFO-41 is a questionnaire developed by the European Osteoporosis Foundation to assess the health-related quality of life of osteoporosis patients. The scale is specifically designed for osteoporosis patients with vertebral fractures and is effective in differentiating health-related  quality of life in patients with and without vertebral fractures. |

TUG: Timed Up and Go; BBS: Berg Balance Scale; FRT: Functional Reach Test; ECOS-16: Escala de Calidad de Vida Osteoporosis; Qualeffo-41 questionnaire : Quality of Life Questionnaire of the European Foundation for Osteoporosis.

**Supplementary Fig. 1.** Sensitivity analysis of pooled results. A. balance; B. Mobility.


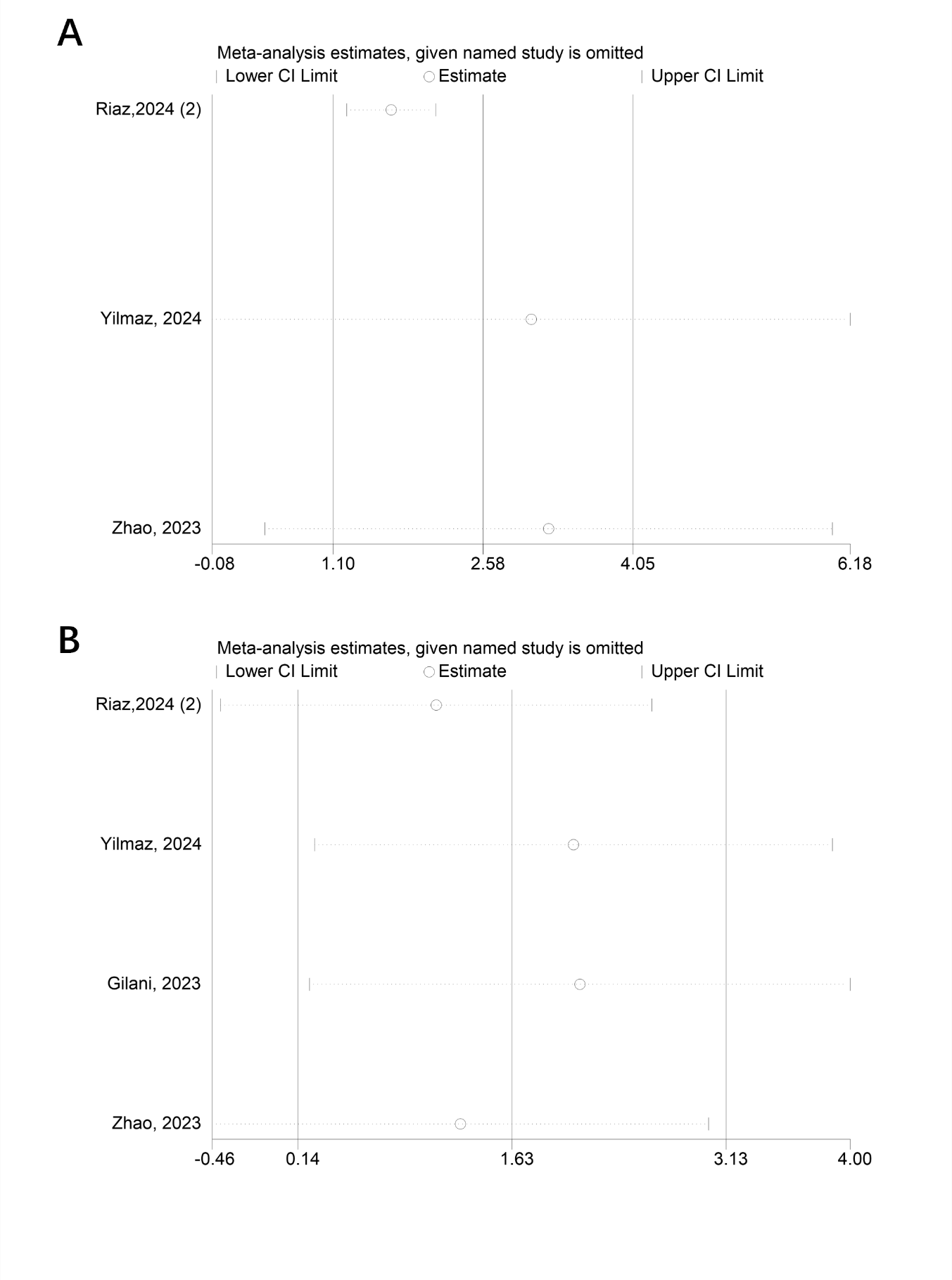


**Supplementary Fig. 2.** Pooled results of subgroup analysis based on interventions of control group. A. balance; B. Mobility

**A**

%

ID SMD(95%CI) Weight

Passive control

Riaz,2024 (2)

Sublotal (I-squared=.%, p = .)

4.69 (3.51, 5.87)

4.69 (3.51, 5.87)

-<>

30.08

30.08


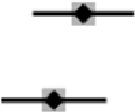


Subtotal (I-squared= 0.0%, p = 0.429)

Overall (I-squared= 91.2%, p = 0.000)

NOTE: Weights **are** from random effects analysis

- 1.67 (1.24, 2.11)


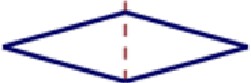
2.58 (1.10, 4.05)

| Active control |  | |
| --- | --- | --- |
| Yilmaz. 2024 | **1.84** (1.24, 2.45) | 35.04 |
| znao, 2023 | 1.49 (0.86, 2.12) | 34.88 |
|  |  | 69.92 |

100.00

-5.87 0 5.87

B

Study ID


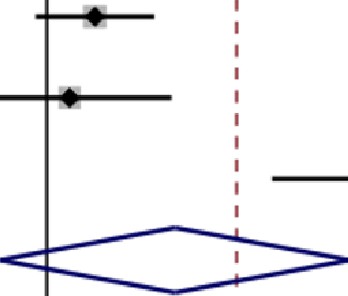


%

SMO (95%CI) Weight

Passive control Riaz,2024 (2)

Subtotal (I-squared = %, p = .)

3.28 (2.35, 4.21)

3.28(2.35,4.21)

-<>

24.34

24.34

| Active control |  | |
| --- | --- | --- |
| Yilmaz, 2024 | 0.41 (-0.10, 0.93) | 26.04 |
| Gilani, 2023 | 0.20 (-0.68, 1.07) | 24.58 |
| Zhao. 2023 | 2.72 (1.94, 3.49) | 25.04 |
| Subtotal (I-squared = 92.6%, p = 0.000) | 1.10 (-0.40, 2.61) | 75.66 |

Overall (I-squared= 93.7%, p = 0.000)

NOTE: Weights are from random effects analysis

1.63 (0.14, 3.13)

100.00


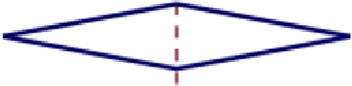
-4.21 0 4.21
